# Bortezomib and Arsenic Trioxide Activity on a Myelodysplastic Cell Line (P39): A Gene Expression Study

**DOI:** 10.4274/tjh.2014.0058

**Published:** 2015-08-01

**Authors:** Hakan Savlı, Sara Galimberti, Deniz Sünnetçi, Martina Canestraro, Giuseppe Palumbo, Balint Nagy, Francesco Di Raimondo, Mario Petrini

**Affiliations:** 1 Kocaeli University Faculty of Medicine, Department of Medical Genetics, Kocaeli, Turkey; 2 Pisa University Faculty of Medicine, Department of Clinical and Experimental Medicine, Division of Hematology, Pisa, Italy; 3 Catania University Faculty of Medicine, Department of Clinical and Molecular Bio-Medicine, Division of Hematology, Catania, Italy; 4 Semmelweis University Faculty of Medicine, Department of Obstetrics and Gynecology, Budapest, Hungary

**Keywords:** Bortezomib, Arsenic trioxide, NF-κB, MDS, Gene expression

## Abstract

**Objective:**

We aimed to understand the molecular pathways affected by bortezomib and arsenic trioxide treatment on myelomonocytoid cell line P39.

**Materials and Methods:**

Oligonucleotide microarray platforms were used for gene expression and pathway analysis. Confirmation studies were performed using quantitative real time PCR.

**Results:**

Bortezomib treatment has shown upregulated DIABLO and NF-κBIB (a NF-κB inhibitor) and downregulated NF-κB1, NF-κB2, and BIRC1 gene expressions. Combination treatment of the two compounds showed gene expression deregulations in concordance by the results of single bortezomib treatment. Especially, P53 was a pathway more significantly modified and a gene network centralized around the beta estradiol gene. Beta estradiol, BRCA2, and FOXA1 genes were remarkable deregulations in our findings.

**Conclusion:**

Results support the suggestions about possible use of proteasome inhibitors in the treatment of high-risk myelodysplastic syndrome (MDS). NF-κB was observed as an important modulator in leukemic transformation of MDS.

## Abstract (tr)

**Amaç:**

Bu çalışmada, bir myelomonositoid hücre hattı olan P39 üzerinde bortezomib ve arsenik trioksidin etkilediği moleküler altyolları anlamayı amaçladık.

**Gereç ve Yöntemler:**

Gen anlatım ve altyol analizleri için oligonükleotid mikroarray platformları kullanıldı. Konfirmasyon deneyleri kantitatif gerçek zamanlı PZR ile gerçekleştirildi.

**Bulgular:**

Bortezomib işlenmesi, DIABLO ve bir NF-κB inhibitorü olan NF-κBIB’nin anlatım artışını ve NF-κB1, NF-κB2, BIRC1 genlerinin anlatım azalışını gösterdi. İki bileşiğin birlikte işlenmesi ise aynı genlerin anlatım düzensizliğini göstererek bortezomib’in tek başına işlenmesinin sonuçlarıyla uyum içindeydi. Özellikle, P53 hücre altyolu daha anlamlı bir değişikliğe uğradı ve beta estradiol geni çevresinde bir gen ağı biçimlendi. Beta estradiol, BRCA2 ve FOXA1 genlerinin düzen değişiklikleri bulgularımız içinde en dikkat çekicileriydi.

**Sonuç:**

Sonuçlar proteazom inhibitörlerinin yüksek riskli myelodisplastik sendromda (MDS) olası kullanımı düşüncesini desteklemektedir. NF-κB, MDS’nin lösemik transformasyonunda önemli bir düzenleyici olarak gözlenmiştir.

## INTRODUCTION

NF-κB is defined as an important transcription factor in immunity, cell survival, and cancer [1,2,3]. NF-κB gene activation was observed in many steps such as tumor progression and metastasis [4,5]. Relationships between NF-κB and leukemia have recently been identified through new mutations on chronic lymphocytic leukemia and specific NF-κB pathway activation of multiple myeloma [6,7]. NF-κB /Rel-blocking approaches have been proposed as antineoplastic strategies. Furthermore, the identification of specific kinases within the NF-κB activation pathway offers a selective target to address tailored therapies. Recent data provided a rationale for therapeutic approaches, which combined different NF-κB inhibitors in chronic myeloid leukemia patients [8]. NF-kB is also a nuclear factor of kappa light polypeptide gene enhancer in B-cells inhibitor and it has been reported to be constitutively activated in the myelomonocytoid cell line P39 [9]. Some MDS subtypes have a high risk of developing into acute myeloid leukemia [10]. Another gene whose expression levels have been reported to play a relevant prognostic role in MDS is WT1. Changes in the expression of the WT1 gene are associated with certain types of lung, prostate, breast, and ovarian cancer. Abnormal expression of the WT1 gene also occurs in leukemia. It is unclear what role the WT1 protein plays in the development or progression of cancer [11]. We decided to assess if a compound combination (bortezomib and arsenic trioxide) able to inactivate NF-kB would be also able to down-regulate the WT1 expression. Finally, we performed microarray and real-time quantitative PCR assays to understand the gene expression pathways affected by this treatment.

## MATERIALS AND METHODS

P39 cell line (DSMZ, Zellkulturen, Braunschweig, Germany) was grown within 48 hours in RPMI 1640 medium (Gibco-LT, CA, USA) under the treatment of different concentrations of bortezomib and arsenic trioxide (ATO) as previously described in our studies [12]. Cell viability was determined by trypan blue exclusion assay, and proliferative responses were assayed by a colorimetric test based on methyl thiazoletetrazolium bromide reduction [13]. After drug exposure, signs of apoptosis were evaluated by light microscopy and the Annexin V/propidiumcytofluorimetric analysis. Reactive oxygen species (ROS) production was evaluated by dihydrorhodamine 123 (DHR) (Molecular Probes, Eugene, OR, USA) and flow-cytometry assay [12]. Samples were also evaluated by the Human Apoptosis Panel (TaqMan®, Applera, Norwalk, USA). All the experiments were repeated at least three times. Reported values represent the means ± SD. The significance of differences between experimental conditions was determined using the 2-tailed Student’s t test. The level of significance was p<0.05. All above cell studies were performed in our laboratories, located at Pisa University. Microarray studies and real time PCR confirmations were performed at Kocaeli University. Microarray analysis was performed using the Whole Human Genome Oligo Microarray (Agilent Technologies), encompassing more than 44,000 human DNA probes. The full list of cDNAs is available online (www.agilent.com). Protocols for sample preparation and hybridization of the mononuclear cells were adaptations of those in the Agilent Technical Manual. In short, first strand cDNA was transcribed from 300 ng of total RNA using T7-Oligo (dT) Promoter Primer. Samples were transcribed in vitro and Cy-3-labeled by using a Quick-AMP labeling kit (Agilent Technologies). Following a further clean-up round (Qiagen), cRNA was fragmented into pieces ranging from 35 to 200 bases in size. Fragmented cRNA samples (1.65 ug) were hybridized onto chips by means of 17 h of incubation at 65 °C with constant rotation, followed by a two-step microarray wash of 1 min in two washing buffers (Agilent Technologies). Hybridized microarrays were scanned in a Agilent Technologies Scanner (model G2505B) and numerical results were extracted with Feature Extraction version 9.5.1.1 using 014850_D_F_20060807 grid, GE1-v5_95_Feb07 protocol and GE1_QCM_Feb07 QC metric set. The microarray data were analyzed using Gene Spring software version 6.1 (Agilent Technologies, Santa Clara, CA, USA). The fold changes were analyzed by filtering the dataset using p-value <0.01 and a signal-to-noise ratio >2 for use in t-test statistical analysis. Additional filtering (minimum 2-fold change) was applied to extract the most of these genes, which were analyzed using Ingenuity Pathway Analysis (IPA) software (Ingenuity Systems, Redwood City, CA, USA). Those genes with known gene symbols (HUGO) and their corresponding expression values were uploaded into the software. Each gene symbol was mapped to its corresponding gene object in the Ingenuity Pathways Knowledge Base. Networks of these genes were algorithmically generated based on their connectivity and assigned a score. The score is a numerical value used to rank networks according to how relevant they are to the genes in the input dataset but may not be an indication of the quality or significance of the network. The score takes into account the number of focus genes in the network and the size of the network to approximate how relevant this network is to the original list of focus genes. The network identified is then presented as a graph indicating the molecular relationships between genes/gene products. Genes are represented as nodes, and the biological relationship between two nodes is represented as an edge (line). The intensity of the node color indicated the degree of up- or down-regulation. The node shapes are disclosed in corresponding figure legends. Canonical pathway analysis identified the pathways from the IPA library of canonical pathways, which were most significant to the input data set. The significance of the association between the data set and the canonical pathway was determined based on two parameters: (1) A ratio of the number of genes from the data set that map to the pathway divided by the total number of genes that map to the canonical pathway and (2) a p-value calculated using Fischer’s exact test determining the probability that the association between the genes in the data set and the canonical pathway is due to chance alone. Real time PCR confirmations were performed as described before [12,14].

## RESULTS

**Figure 1:**
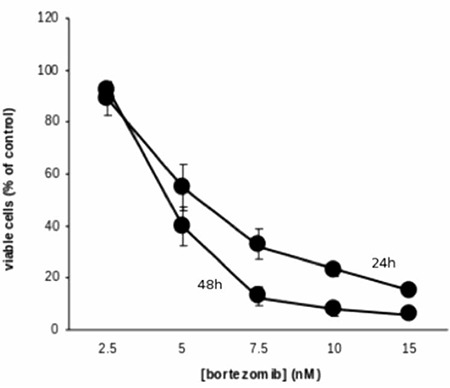
Bortezomib exerted a significant anti-proliferative effect in a dose and time-dependent manner.

**Figure 2:**
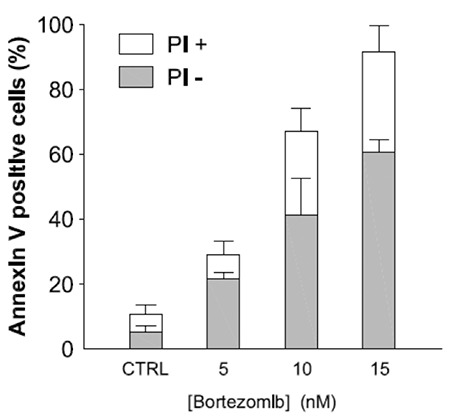
A significant increase of cell apoptotic rate was observed after treatment with the association of bortezomib (p<0.05, Fisher’s exact test). Columns, means of separate experiments; bars, standard deviation. Bortezomib exerted a significant pro-apoptotic effect (Annexin-V assay) after 48 hours incubation period. First column (CTRL) indicates the control sample without bortezomib.

**Figure 3:**
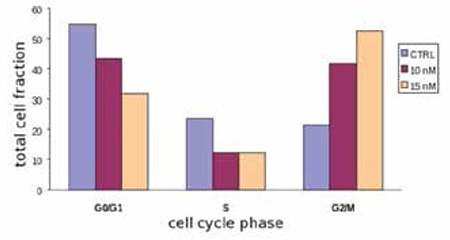
Flow cytometry analysis of accumulation of cells with DNA content. The apoptotic cell fraction increase required the simultaneous presence of bortezomib (∗p<0.05, Fisher’s exact test). Columns, means of three separate experiments; bars, SD. Bortezomib induces a cell cycle block in G2 phase. First columns indicate the (CTRL) control samples without bortezomib.

**Figure 4:**
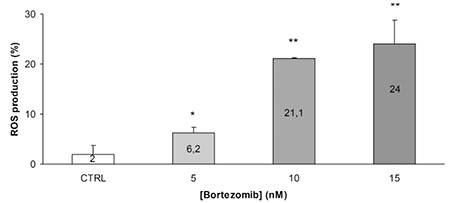
ROS production after bortezomib treatment. Exposure of cells with and without bortezomib after which they were labeled with dihydrorhodamine 123 and analyzed by flow cytometry, to determine the percentage of cells displaying an increase in reactive oxygen species production (∗p<0.05 with respect to control; #p<0.05 with respect to bortezomib alone; Fisher’s exact test). Columns, means of at least three separate experiments; bars, SD. (b) Role of ROS in ATO/bortezomib-mediated lethality in HL60 cells. Bortezomib induces a significant ROS production after 48 hours incubation period. First column (CTRL) indicates the control sample without bortezomib.

**Figure 5:**
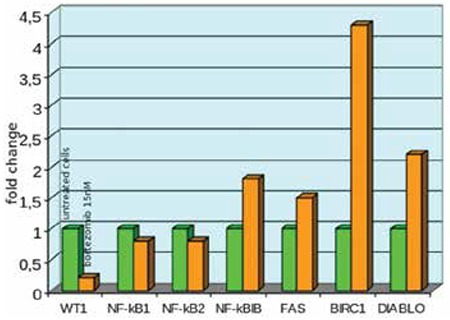
WT1 gene expression was observed as most down-regulated, and BIRC1 gene expression was observed as most up-regulated. NF-kB gene was down-regulated while its inhibitor NF-kB1B gene was up-regulated. Results obtained after 48 hours incubation period.

Bortezomib inactivated NF-kB and exerted an anti-proliferative (Figure 1) and pro-apoptotic effect (Figure 2) by blocking cell cycle in the G2 phase (Figure 3). It increased the release of reactive oxygen species (Figure 4) and down-regulated the WT1 expression (Figure 5). In the untreated P39, 84 of the 93 genes involved in the apoptosis pathway and representation in the Taqman Low-Density Arrays were expressed. After treatment, bortezomib had up-regulated DIABLO and NFkBIB (NF-kB inhibitor), and down-regulated the NF-kB1, NF-kB2, and BIRC1, an anti-apoptotic gene (Figure 5). Seven gene pathways (P53, PPAR, IL6, IL2, hypoxia, Huntington’s disease, TLR) were found most significantly de-regulated in our microarray analysis.

**Figure 6:**
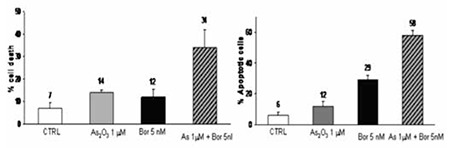
Bortezomib and arsenic trioxide exert synergistic anti-proliferative and pro-apoptotic effects.

**Figure 7:**
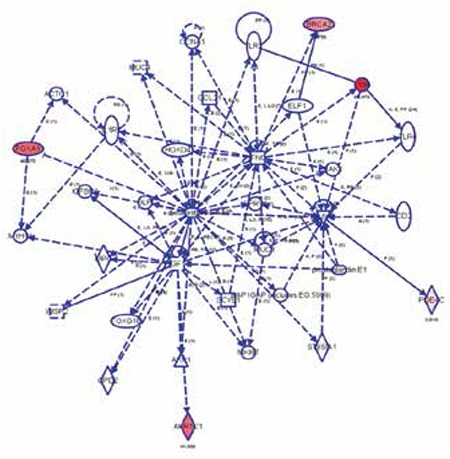
Significant up and down regulated gene network identified around genes after bortesomib+arsenic trioxide treatment for 48 hours to P39 cell line.

Bortezomib and ATO combination resulted in synergistic anti-proliferative and pro-apoptotic effects (Figure 6). PPAR, P53, IL6, IL2, hypoxia, Huntington’s disease, TLR and cell cycle were the pathways more significantly modified in the the IPA analysis of our data, when P39 was co-incubated with bortezomib/ATO. Moreover, we observed SHC1, MLL, ITGAV, BCRA2, HMOX1, ICAM1, JUN, PMAP1 and beta estradiol genes were down-regulated whereas BRCA2, FOXA1, LY96 and AKR1C1 genes were up-regulated. Levels and inter relationships were defined in Figure 7.

## DISCUSSION

We have previously detected the over expressed levels of NF-κB gene in our microarray screening studies on prostate cancer, papillary thyroid cancer and leukemia [12,14,15,16,17]. This study is our first observation about the relationship between dysregulated NF-kB expression levels and MDS.

P53 is a well-known cell cycle regulator and two studies have shown the relationship between NF-kB gene expression and lymphoblastic leukemia pathogenesis before [18,19]. Bastian et al. observed an activation of NF-κB after bortezomib treatment and the induction of apoptosis-related NF-κB target genes such as TNFαRs after concomitant treatment, indicating a possible involvement of NF-κB as a proapoptotic mediator. These findings are not in concordance with ours about the bortezomib induction in a myelodysplastic cell line (P39). It is possible that bortezomib activity has different trigger effects to NF-κB deregulation on different types of leukemic cells.

Among the down-regulated genes, JUN, HSP70 and HSP90 would be relevant. Indeed, high levels of HSPs have been reported to negatively condition the overall survival of patients with high-risk MDS [20]. Other down-regulated genes were CREBBP, PMAIP1, SPP1, and some adhesion molecules, such as ICAM1. Even reduction of integrins could be a relevant effect exerted by the proteasome inhibitor due to higher serum levels of ICAM1 that have been observed to be associated with high-risk MDS, negatively conditioning the survival [21].

Recently, Phase 1 studies indicated that bortezomib is a well-tolerated treatment option in acute myeloid leukemia [22]. Further studies should focus on the role of NF-kB and other pathways as key regulators to therapeutic effect of bortezomib in acute myeloid leukemia. Authors indicated that intracellular NAD* level represents a major determinant in the ability of bortezomib to induce apoptosis [23]. It is still unknown as to how many more pathways and gene networks exist on these apoptotic effects.

Combinations of our two compounds showed gene expression deregulations which were in concordance with the bortezomib treatment alone. Especially, P53 was a pathway more significantly modified and same seven gene pathways were most significantly de-regulated. JUN and ICAM1 genes were found down-regulated again.

Beta estradiol, BRCA2 and FOXA1 genes were remarkable in our findings. ATO and bortezomib combination resulted in a gene network centralized around the beta estradiol gene. It has been previously reported that when estrogen binds its receptor it becomes a potent mitogen in the pathogenesis of mammary carcinoma and ATO specifically inhibits expression and signaling pathway of these receptor [24]. Here we can see the ATO’s downregulation effect over this gene. Besides, protein product of beta-estradiol leads to granulocytic differentiation when itself used as a compound on human CD34+ cells [25]. We believe that the gene network around beta estradiol is important to understand relationship about differentiation treatment by ATO on AML pathogenesis.

BRCA2 has been defined as a part of important pathway to prevent a subgroup of human leukemias and lymphomas that may involve non-random, characteristic gene rearrangements [26]. Upregulated levels of this pathway by the treatment of our compounds was an expected result. However, upregulated levels were extremely high as 29-fold. We found a relationship between BRCA2 and beta estradiol genes over FNG gene downregulation. FNG (Fringe) is a regulator of communication between lineage compartments during hematopoietic development [27]. We found the down regulated levels of beta estradiol were in a signal communication by BRCA2 upregulation. It means that bortezomib and ATO exposure is effective on different anti-cancer mechanisms as ‘DNA repair’ or ‘cell differentiation’, in P39 cell line.

Importance of FOXA1 gene was defined before in cell transcription in different mutations in acute myeloid leukemia and also offered as a promising prognostic marker in breast cancer [28,29]. Upregulated level of this gene was remarkable deregulation in our experiment.

It is possible to discuss similarities and relations between NF-kB and these three genes mentioned above. NF-kB is an important transciption factor as FOXA1 and its critical role is well defined in multiple myeloma [30]. Homolog recombinations in DNA repair induction by NF-κB required the target BRCA2 [31]. Estrogen modulates NFeq signaling at the promoters of inflammatory genes via estrogen receptor-B [32]. We have used compounds effective to inactivate NF-kB and these affection looks deeper when we add ATO to bortezomib.

Taken together, NF-kB was observed as an important modulator in leukemic transformation of MDS. These results suggest the possible use of proteasome inhibitors in treatment of high-risk MDS. In vivo trials will be useful to confirm this hypothesis. Moreover, our study was a preliminary observation of a cell line exposed to drug combinations and different leukemic cells should be studied in this manner as many dimensions of the studies would be discussed separately. This would create insight to the arising gene pathways after bortezomib and ATO exposure in leukemia, and their specific traces on networks defined on miRNA investigation and proteomic levels.
